# Total isostatic response to the complete unloading of the Greenland and Antarctic Ice Sheets

**DOI:** 10.1038/s41598-022-15440-y

**Published:** 2022-07-06

**Authors:** Guy J. G. Paxman, Jacqueline Austermann, Andrew Hollyday

**Affiliations:** 1 Lamont-Doherty Earth Observatory of Columbia University, Palisades, NY 10964 USA; 2 Department of Geography, Durham University, Durham, DH1 3LE UK

**Keywords:** Geophysics, Cryospheric science

## Abstract

The land surface beneath the Greenland and Antarctic Ice Sheets is isostatically suppressed by the mass of the overlying ice. Accurate computation of the land elevation in the absence of ice is important when considering, for example, regional geodynamics, geomorphology, and ice sheet behaviour. Here, we use contemporary compilations of ice thickness and lithospheric effective elastic thickness to calculate the fully re-equilibrated isostatic response of the solid Earth to the complete removal of the Greenland and Antarctic Ice Sheets. We use an elastic plate flexure model to compute the isostatic response to the unloading of the modern ice sheet loads, and a self-gravitating viscoelastic Earth model to make an adjustment for the remaining isostatic disequilibrium driven by ice mass loss since the Last Glacial Maximum. Feedbacks arising from water loading in areas situated below sea level after ice sheet removal are also taken into account. In addition, we quantify the uncertainties in the total isostatic response associated with a range of elastic and viscoelastic Earth properties. We find that the maximum change in bed elevation following full re-equilibration occurs over the centre of the landmasses and is +783 m in Greenland and +936 m in Antarctica. By contrast, areas around the ice margins experience up to 123 m of lowering due to a combination of sea level rise, peripheral bulge collapse, and water loading. The computed isostatic response fields are openly accessible and have a number of applications for studying regional geodynamics, landscape evolution, cryosphere dynamics, and relative sea level change.

## Introduction

The Greenland and Antarctic Ice Sheets constitute significant loads that isostatically depress the Earth’s land surface by amplitudes of hundreds of metres over wavelengths of thousands of kilometres. Understanding the response of the solid Earth, global gravity field, and rotation axis to changes in ice and ocean loading (‘glacial isostatic adjustment’) is central to the aim of constraining past, present, and future changes in ice sheet behaviour and relative sea level^1^. An important end-member scenario to consider is the total isostatic deformation of the Earth’s solid surface, and resulting change in bed topography, that would arise from the removal of the entire Greenland and Antarctic Ice Sheets.

Accurate computation of the fully-rebounded topography of Greenland and Antarctica is important when considering (e.g.): (i) likely nucleation sites of early ice caps during glacial inception^2,3^, (ii) the extent of bed below sea level, seaway connectivity, and marine sediment deposition under ice-free conditions^4^, (iii) the routing of water and formation of lakes on a pre-glacial landscape^5^, (iv) interpretations of subglacial geomorphology^6,7^, and (v) the relationship between regional geodynamics, tectonic structure, topography, and ice dynamics^8,9^.

Although a number of studies have computed isostatically rebounded digital elevation models for an ice-free Greenland or Antarctica^5,10–12^, these products are often not readily accessible and/or utilise ice thickness compilations that have been superseded as data coverage has increased^13^. They also commonly employ simplified isostatic models with a uniform zero (Airy isostasy) or non-zero flexural rigidity, which fail to capture lateral variations in Earth structure across Greenland and Antarctica. Here, we use contemporary, freely available, compilations of ice thickness and Earth structure to develop isostatic adjustment grids for a fully re-equilibrated, ice-free, Greenland and Antarctica. This new gridded data product can be readily combined with present-day bed elevation measurements to determine the rebounded topography of any region of interest under ice-free conditions.

## Data

### Grounded ice thickness and bed topography

**Figure 1:**
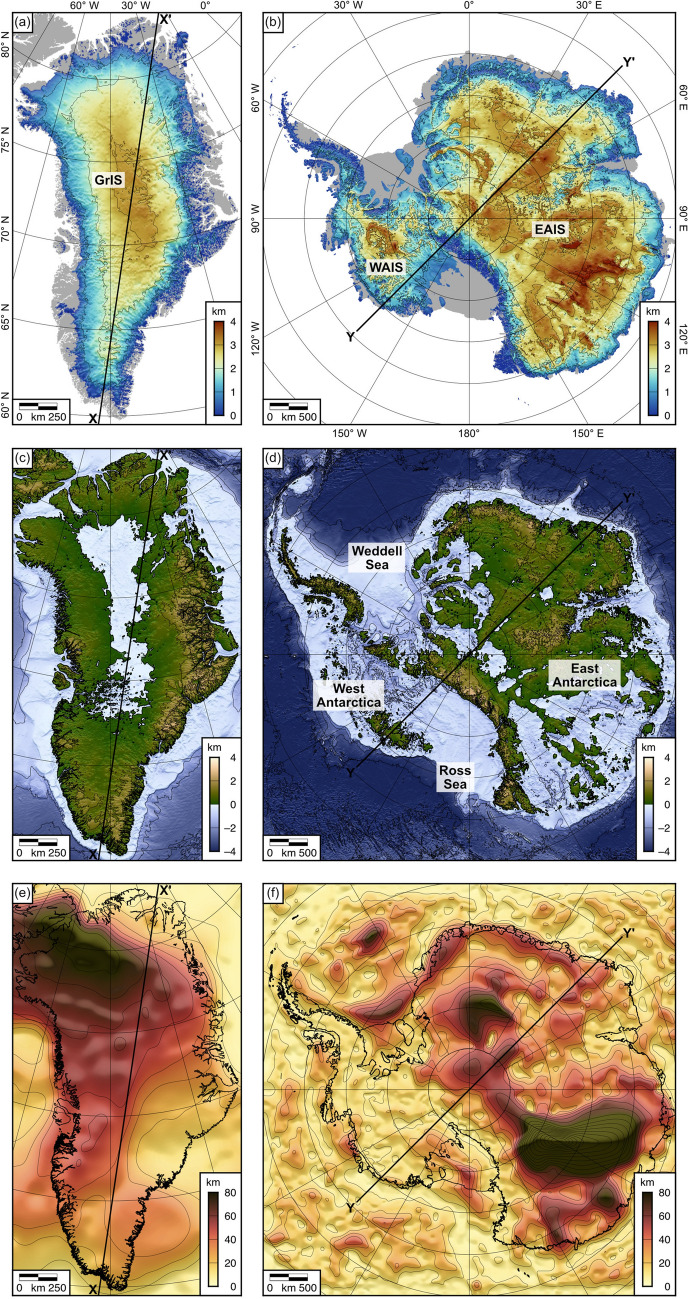
Ice load and effective elastic thickness for Greenland and Antarctica. (**a**) Grounded ice thickness of the modern Greenland Ice Sheet (GrIS)^14^. (**b**) Grounded ice thickness of the modern Antarctic Ice Sheet (EAIS = East Antarctic Ice Sheet; WAIS = West Antarctic Ice Sheet)^15^. Contour interval is 1 km. Grey areas denote ice-free land and floating ice shelves. (**c**) Present-day Greenland bed topography^14^. (**d**) Present-day Antarctic bed topography^15^. Contour interval is 1 km. (**e**) Effective elastic thickness (*T*_*e*_) model for Greenland^16^. (**f**) *T*_*e*_ model for Antarctica^17^. Contour interval is 10 km. Figure created using Generic Mapping Tools v.6 (https://www.generic-mapping-tools.org)^18^.

The modern-day ice sheet loads and bed topographies were acquired from the self-consistent BedMachine Antarctica and Greenland compilations^14,15^ (Fig. 1a–d). BedMachine derives bed elevation and ice thickness using a combination of mass conservation in regions of fast ice flow along the periphery of the ice sheet, and kriging and/or streamline diffusion to interpolate between radio-echo sounding line data in the slow-moving interior. Since it is only grounded ice (as opposed to floating ice) that loads and causes isostatic subsidence of the land surface, we masked the ice thickness grids to set the thickness of the ice load outside the grounding line to zero (Fig. 1a,b). We used the most recently released versions of BedMachine available at the time of writing (version 4 for Greenland and version 2 for Antarctica). The nominal resolution of the BedMachine grid is 150 m in Greenland^14^, and 500 m in Antarctica^15^, although the true resolution varies depending on the coverage of the raw data, with the separation between measurements as large as 100 km in the least accessible parts of the ice sheet interior.

### Effective elastic thickness of the lithosphere

The effective elastic thickness (*T*_*e*_) is a proxy for the depth-integrated strength of the lithosphere^19^, and is a measure of the ability of the lithosphere to support (sub-)surface loads over geological timescales via flexural isostasy^20^. *T*_*e*_ varies significantly on Earth’s continents, with values ranging from 0 to 200 km^21^, and appears to depend on thermomechanical properties of the lithosphere such as age, thermal history, composition, and state of stress^22^. Reliable estimates of *T*_*e*_ have historically been difficult to establish in the polar regions, given the sparse coverage of geological and geophysical observations and poorly constrained crustal structure. However, we used laterally variable *T*_*e*_ grids that have recently been derived using continental-scale spectral analysis of gravity and topography data in Antarctica^17^ and Greenland^16^ (Fig. 1e,f).

## Methods

### Modern ice sheet unloading

To model the response of the solid Earth to the removal of the modern ice sheets, we assumed that the lithosphere and mantle have attained long-term isostatic equilibrium following full deglaciation. We assumed that transient viscoelastic mantle responses have completely relaxed, which would typically be achieved over timescales on the order of 100 kyr^23^. In this case, where we are concerned with the fully re-equilibrated steady-state of the lithosphere on timescales that greatly exceed the relaxation timescale, the isostatic response of the Earth approximates that of a flexed elastic plate (i.e., the lithosphere) above an inviscid substrate (i.e., the underlying mantle)^24^. We therefore used an elastic plate model to determine the vertical displacement resulting from the unloading of the modern ice sheets. The general 2D flexure equation is expressed as^20^:

1 $${\nabla }^{2}\left[D\left(x,y\right){\nabla }^{2}w\left(x,y\right)\right]+\left({\rho }_{mantle}-{\rho }_{infill}\right)gw\left(x,y\right)=({\rho }_{load}-{\rho }_{displace})gh\left(x,y\right)$$

where

2 $$D\left(x,y\right)=\frac{E{T}_{e}^{3}(x,y)}{12(1-{\nu }^{2})}$$

is the flexural rigidity of the lithosphere as a function of spatial dimensions *x* and *y*. *E* (Young’s modulus; 100 GPa) and *ν* (Poisson’s ratio; 0.25) are elastic constants, and *g* is the gravitational acceleration (9.81 m s^–2^). The load is denoted by *h*, and the flexural deformation is given by *w*. We assumed standard ice load (*ρ*_*load*_) and mantle (*ρ*_*mantle*_) densities of 917 and 3330 kg m^–3^, respectively. The *ρ*_*displace*_ and *ρ*_*infill*_ terms refer to the density of the material displaced by the load and infilling the flexural moat, respectively. In this study, the displaced/infilling material is either air (0 kg m^–3^) or seawater (1028 kg m^–3^), depending on the situation being considered.

We solved Eq. (1) for the laterally variable *T*_*e*_ scenarios of Steffen et al. (2018)^16^ and Swain & Kirby (2021)^17^ using a centred finite difference technique^25^. Steffen et al. (2018) place uncertainties of up to 40 km on the spectral *T*_*e*_ estimates for Greenland, but uncertainties are not quantified for the Antarctic estimates. To determine a simplified measure of the uncertainty in the flexure, we also solved Eq. (1) for three uniform *T*_*e*_ values: 10, 40, and 70 km. These values were selected to cover the range between the lower and uppermost commonly observed values (with the exception of localised maxima; Fig. 1e,f). In assessing the uncertainty in the calculated flexure, each of the three uniform *T*_*e*_ scenarios and the spatially variable scenario were given equal weighting. When *T*_*e*_ is spatially uniform, Eq. (1) can be solved analytically via a fast Fourier transform of the load and convolution with a 2D flexural isostatic response function^20^. These uniform *T*_*e*_ calculations were also used to validate the numerical solutions for the laterally variable *T*_*e*_ scenarios.

In the interest of computational efficiency, calculations were performed at a horizontal resolution of 5 km, and the resulting isostatic deformation grids were subsequently resampled to match the spatial resolution and extent of the BedMachine ice thickness and bed elevation models^14,15^.

### Last Glacial Maximum disequilibrium

In addition to the deformation induced by modern ice load removal, we applied a correction for the ongoing unequilibrated response of the solid Earth to the loss of ice since the Last Glacial Maximum (LGM). Because we sought to determine the residual part of the post-LGM isostatic response that has not yet equilibrated, we calculated the time-dependent viscoelastic response that is still to come due to the LGM-to-present ice load change using a self-gravitating viscous Earth model, following the approach described in ref^26^. We constructed an ice load history from the Eemian (122 ka) to the present-day. We used a eustatic sea level curve to capture the growth of ice up to the LGM (26 ka)^27^, and the ICE-6G_C model for subsequent deglaciation^28^. We calculated the residual disequilibrium in the solid Earth deformation and the geoid (sea surface) height change (which is caused by shifts in the distribution of mass within the Earth).

To allow for uncertainties in the viscoelastic structure of the mantle beneath Antarctica and Greenland, the unequilibrated solid surface deformation and geoid change were assumed to be the mean responses of a suite of viscoelastic Earth models, with 24 combinations of lower mantle viscosity (0.3, 0.5, 0.7, 1.0, 2.0, and 3.0 × 10^22^ Pa s), upper mantle viscosity (3.0 and 5.0 × 10^20^ Pa s), and elastic lithosphere thickness (71 and 96 km). These ranges of values reflect typical spatially-averaged estimates for the entirety of Greenland and Antarctica^28–31^. Note that this lithosphere thickness is not equivalent to *T*_*e*_; it instead describes the thickness of an idealised elastic layer above the viscoelastic mantle appropriate for ice age timescales^32^. For the above range of mantle viscosities, the timescale required for the residual disequilibrium to decay by a factor of 1/5*e* from its post-LGM peak value and thereby reach approximate steady state varies from 30 to 70 kyr.

Although these reference lithosphere thickness and mantle viscosity values are likely realistic for much of the two regions, we note that upper mantle viscosities up to two orders of magnitude lower have been recovered from certain locations in West Antarctica and eastern Greenland^31,33^. However, in the absence of readily available, robust 3D mantle viscosity estimates, we assume a radial viscosity profile for simplicity and computational efficiency.

### Sea level change and water loading feedback

Following the removal of the ice sheet loads, areas of the rebounded landmasses that remain situated below sea level will be subjected to loading by water that replaces the ice. To calculate the effect of this feedback, we first added the solid Earth deformation triggered by modern ice unloading and post-LGM disequilibrium to the BedMachine bed topographies (Fig. 1c,d). We then calculated the water load geometry in areas below sea level assuming a uniform eustatic sea level rise of 65.3 m caused by the removal of the modern ice sheets (7.42 m from Greenland^14^, 57.9 m from Antarctica^15^) and an additional non-uniform sea surface height change caused by the residual post-LGM re-equilibration of the geoid. We neglected the rotational and gravitational feedbacks associated with loss of mass from the modern ice sheets, because in this case we are moving from one equilibrium state to another (modern ice to no ice) and the gravity change due to ice mass loss is largely cancelled out by the gravity change due to solid Earth deformation. The flexural response to water loading was computed using Eq. (1). Since this water loading in turn modifies the geometry of the coastline and of the water load itself, changes to the water load and the resulting flexure were calculated iteratively until the average load change fell below 1 m, which required up to three iterations.

### Total isostatic response

For the modern ice sheet unloading and post-LGM disequilibrium components, the total isostatic adjustment was defined as the displacement of the Earth’s solid surface with respect to the geoid (sea surface height):

3 $$\Delta T=\Delta R-\Delta G$$

where $\Delta T$ is the change in topography (the opposite of the relative sea level change), $\Delta R$ is the solid surface deformation, and $\Delta G$ is the change in sea surface height. The solid surface deformation is the field computed using the elastic plate model and the self-gravitating viscous Earth model; the change in sea surface height is the eustatic sea level rise for the loss of the modern ice sheets plus the non-uniform as-yet-unequilibrated residual component following LGM-to-present ice loss.

**Figure 2:**
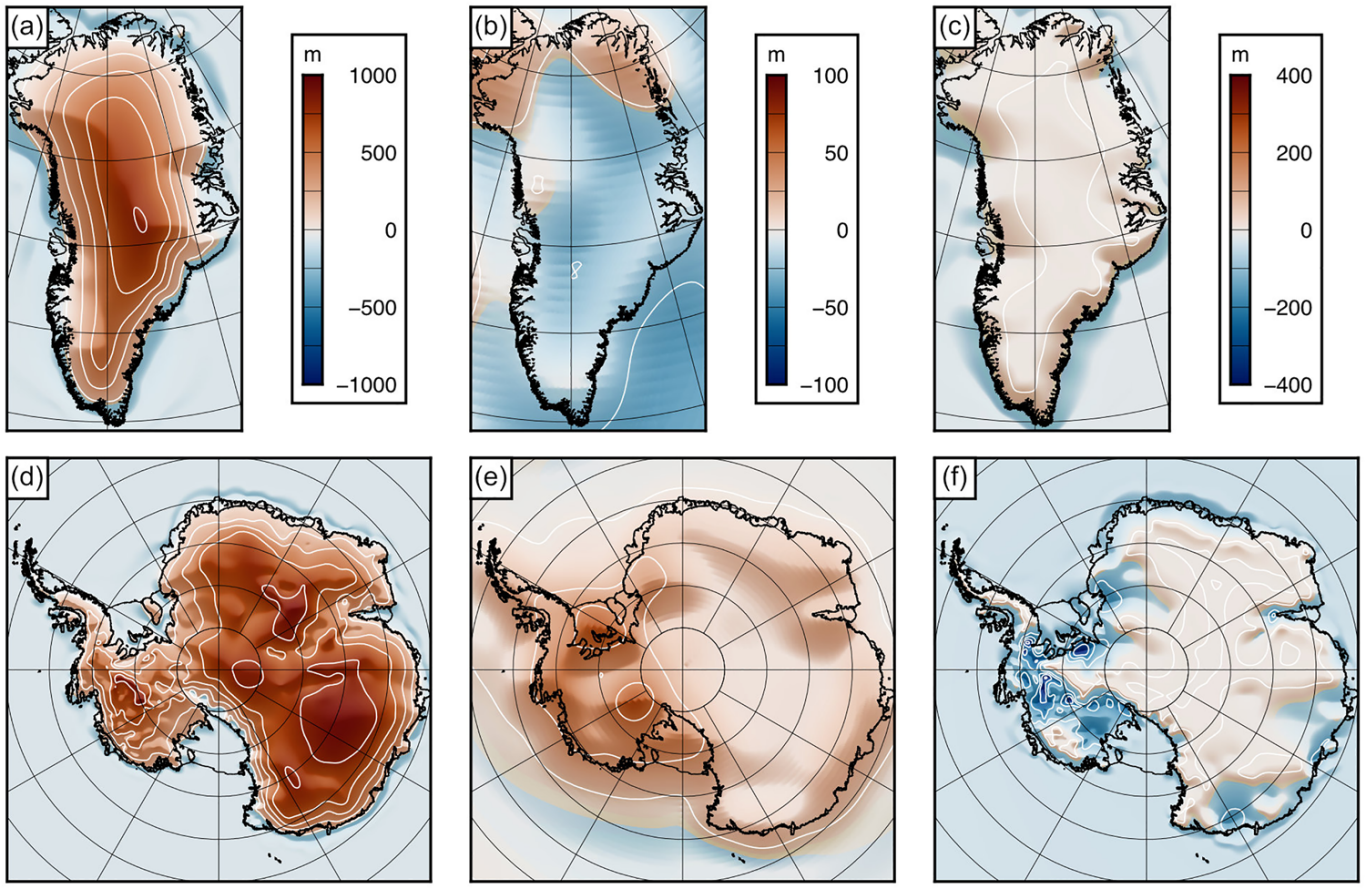
Components of isostatic adjustment. (**a**) Change in bed elevation ($\Delta T$) caused by the unloading of the Greenland Ice Sheet for the laterally variable effective elastic thickness scenario. Contour interval is 200 m. (**b**) Change in bed elevation ($\Delta T$) caused by the remaining post-LGM viscoelastic disequilibrium in Greenland for the mean of the 24-model suite. Contour interval is 25 m. (**c**) Change in bed elevation ($\Delta T$) caused by the water loading feedback in Greenland for the laterally variable effective elastic thickness scenario. Contour interval is 100 m. (**d**), (**e**), (**f**) same as above panels but for Antarctica. Figure created using Generic Mapping Tools v.6 (https://www.generic-mapping-tools.org)^18^.

The contributions of modern ice sheet unloading, post-LGM disequilibrium, and water loading feedback (Fig. 2) were summed to give the total isostatic response for Greenland and Antarctica. To quantify the total uncertainty in the modelled isostatic adjustment, we summed the standard deviation associated with (i) the 24 viscoelastic models used to compute the post-LGM disequilibrium, and (ii) the four elastic models (*T*_*e*_) used to compute the long-term flexural response to the removal of the modern ice load and the water loading feedbacks.

## Results

### Components of isostatic adjustment

The dominant contributor to full-deglacial isostatic adjustment in Antarctica and Greenland is the uplift induced by the removal of the modern ice sheet loads. The rebound driven by modern ice unloading exhibits a concentric pattern well correlated with the ice thickness (Fig. 1a,b), causing a maximum change in elevation of +806 m in central Greenland and +936 m in central East Antarctica for the spatially variable *T*_*e*_ scenarios (Fig. 2a,d; Table 1). A drop in elevation of up to –96 m occurs offshore (Fig. 2a,d) due to a combination of peripheral bulge collapse around the ice sheet margin and eustatic sea level rise.

**Table 1:** Summary statistics for the isostatic response to complete deglaciation in Greenland and Antarctica. Statistics are calculated for the areas of Greenland and Antarctica inside the present-day grounding line. The minimum, maximum, and mean values are spatial statistics calculated for isostatic responses ($\Delta T$) associated with the laterally variable *T*_*e*_ scenarios. The standard deviation is across all Earth models used in this study, including the four *T*_*e*_ models for calculating the modern ice and water load flexure, and the 24 model parameter combinations in the viscoelastic model for calculating the post-LGM disequilibrium.

	Minimum (m)	Maximum (m)	Mean (m)	Standard deviation across all Earth models (spatial average) (m)
**Greenland**				
Modern ice unloading	–96.1	+806	+312	21.4
post-LGM disequilibrium	–25.2	+25.1	–9.26	8.52
Water loading	–19.7	+1.04	–2.31	0.89
Total isostatic response	–116	+783	+301	21.4
**Antarctica**				
Modern ice unloading	–92.9	+936	+508	22.9
post-LGM disequilibrium	–3.91	+68.3	+14.4	5.80
Water loading	–422	+13.8	–27.8	6.77
Total isostatic response	–123	+936	+494	22.1

The contribution from post-LGM disequilibrium is comparatively minor, with the largest resulting shifts in elevation –25 m in Greenland and +68 m in Antarctica (Table 1). Unequilibrated post-LGM uplift in Antarctica is centred on the Weddell Sea and Ross Sea embayments (Fig. 2e), which have experienced appreciable ice sheet retreat and mass loss since the LGM^34^. By contrast, the remaining unequilibrated post-LGM solid Earth response in Greenland is mostly negative (subsidence) (Fig. 2b). This is because Greenland is situated in the collapsing >1000 km-wide peripheral bulge of the former Laurentide Ice Sheet in North America, which has lost approximately one order of magnitude more mass since the LGM than the Greenland Ice Sheet^28^.

East Antarctica and Greenland are almost entirely situated above sea level after modern and post-LGM ice rebounding, so the effect of the water loading feedback is negligible in these regions (Fig. 2c,f). However, low-lying parts of West Antarctica (Fig. 1d) remain situated well below sea level following the removal of the ice load, and subsequent inundation by water induces localised subsidence of up to –422 m (Table 1), which partially offsets the effect of ice unloading.

**Figure 3:**
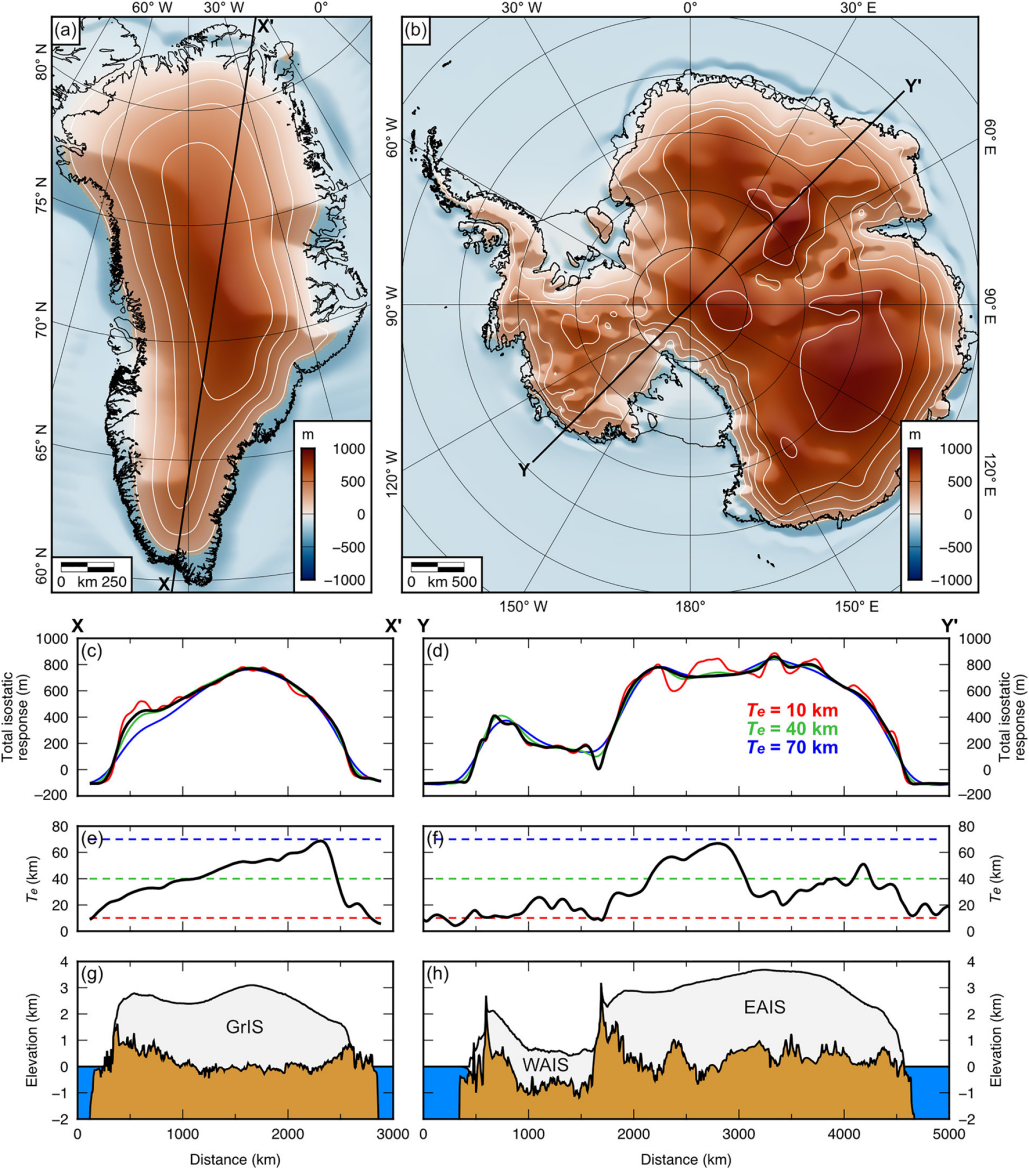
Total isostatic response to the complete unloading of ice from Greenland and Antarctica. (**a**) Total change in Greenland bed elevation ($\Delta {T}$) caused by the isostatic response to full deglaciation (sum of the components displayed in Fig. 2a–c). (**b**) Total change in Antarctic bed elevation ($\Delta {T}$) caused by the isostatic response to full deglaciation (sum of the components displayed in Fig. 2d–f). Contour interval is 200 m. (**c**) Sensitivity of the isostatic deformation to *T*_*e*_ along transect X–X’ of the Greenland Ice Sheet. Black line shows the response for the spatially variable *T*_*e*_ scenario; coloured lines show the response for uniform *T*_*e*_ values of 10 km (red), 40 km (green), and 70 km (blue). (**d**) Sensitivity of the isostatic deformation to *T*_*e*_ along transect Y–Y’ of the Antarctic Ice Sheet. Colour notation is identical to panel c. (**e**) *T*_*e*_ across Greenland. Black line shows the spatially variable *T*_*e*_ scenario^16^, horizontal dashed lines show the three uniform *T*_*e*_ values tested. (**f**) *T*_*e*_ across Antarctica^17^. Colour notation is identical to panel e. (**g**) Greenland Ice Sheet (GrIS) and bed topography^14^. (**h**) West Antarctic Ice Sheet (WAIS), East Antarctic Ice Sheet (EAIS), and bed topography^15^. Elevations are relative to modern-day mean sea level. Figure created using Generic Mapping Tools v.6 (https://www.generic-mapping-tools.org)^18^.

The fully equilibrated isostatic response to complete ice sheet unloading (Fig. 3a,b) is dominated by the effect of modern ice sheet unloading, with secondary contributions from post-LGM disequilibrium and water loading feedbacks. The mean bed elevation changes are +301 m within Greenland and +494 m within Antarctica, with maxima of +783 m and +936 m, respectively (Table 1). The area around the ice margins experiences minor subsidence due to a combination of peripheral bulge collapse and the effects of water loading (Fig. 3a,b).

The isostatic calculations that incorporate a laterally variable *T*_*e*_ model demonstrate that the frequency content of the flexure signal is dependent on *T*_*e*_ (Fig. 3c,d). Shorter wavelength (higher frequency) variability in the flexure is observed in areas of low *T*_*e*_ such as West Antarctica and southern Greenland, whereas longer wavelength signals dominate in areas of high *T*_*e*_ such as East Antarctica and northern Greenland (Fig. 3c–h). Over wavelengths comparable to the length-scale of the ice sheets (>10^3^ km), the pattern of flexure is largely independent of *T*_*e*_ (Fig. 3)^20^. In any particular location, the flexure calculated numerically for the laterally variable *T*_*e*_ scenario is in good agreement with the flexure calculated analytically using the most proximal uniform *T*_*e*_ value (Fig. 3c,d). This indicates that the numerical finite difference approach used to compute the flexure for an elastic plate of spatially variable thickness is accurate and robust.

### Uncertainty estimation

**Figure 4:**
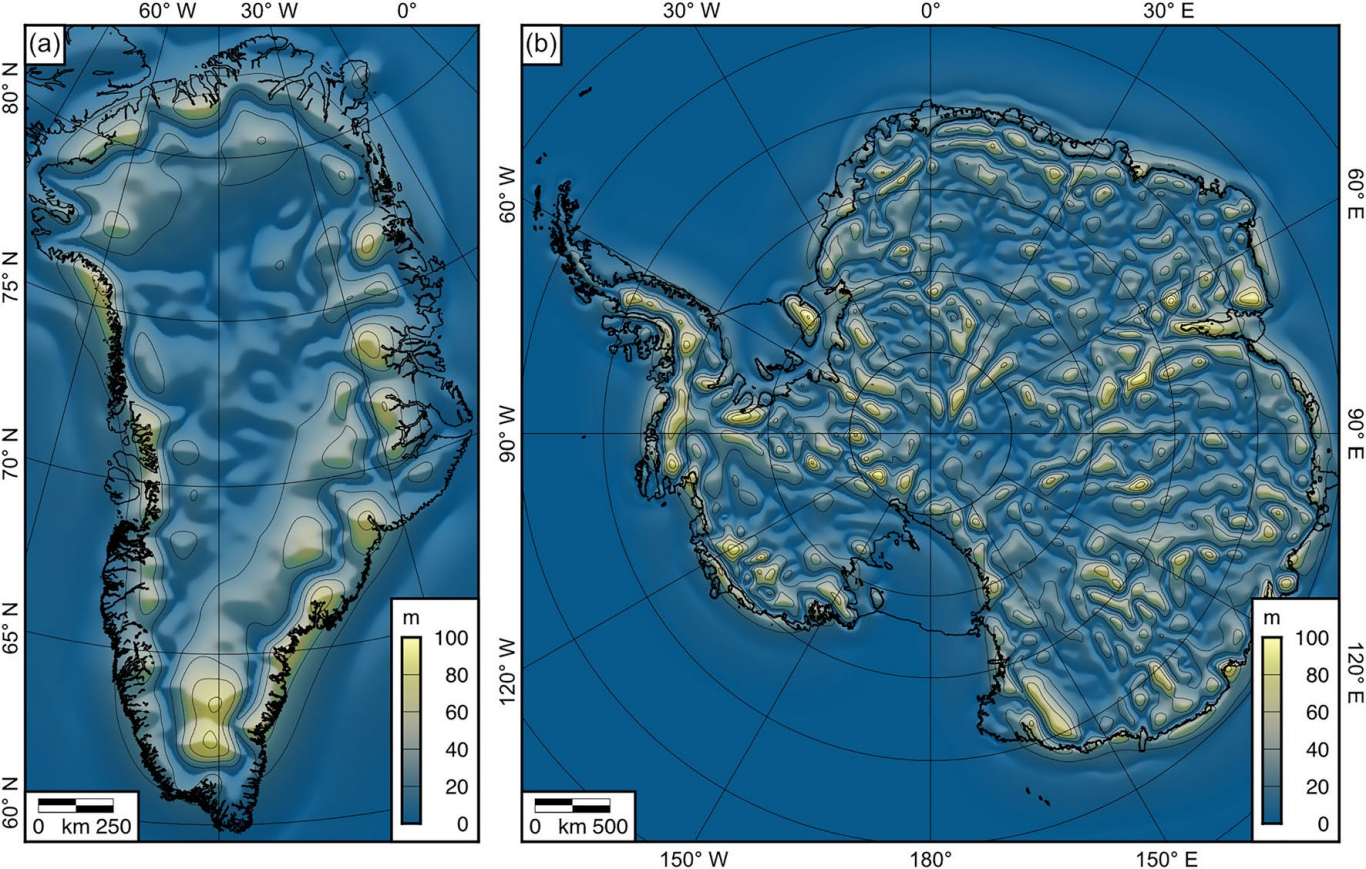
Standard deviation of the total isostatic response to ice sheet removal. (**a**) Standard deviation of the total isostatic response for Greenland. (**b**) Standard deviation of the total isostatic response for Antarctica. The standard deviation is calculated across all Earth models, including the four *T*_*e*_ models for calculating the modern ice and water load flexure, and the 24 parameter combinations in the viscoelastic model for calculating the post-LGM disequilibrium. Contour interval is 20 m. Figure created using Generic Mapping Tools v.6 (https://www.generic-mapping-tools.org)^18^.

We found that the spatially averaged standard deviation in the total isostatic response across the suite of tested Earth models is 21.4 m in Greenland and 22.1 m in Antarctica (Table 1). Standard deviations of up to 100 m are found in regions of large spatial gradients in ice thickness, such as around the ice sheet margins and/or above areas of steep subglacial topography (Fig. 4). The primary source of uncertainty is the *T*_*e*_ model associated with the flexural response to modern ice unloading (Table 1), which is intuitive given that this component comprises the bulk of the total isostatic response (Fig. 2). We note that there is additional uncertainty associated with the assumption of laterally uniform Earth properties in the post-LGM disequilibrium calculation (see "Last Glacial Maximum disequilibrium" Section). Although this uncertainty is difficult to quantify and we do not incorporate it explicitly, the post-LGM component is a comparatively small contributor to the total isostatic response (Fig. 2).

In addition to the uncertainties associated with the isostatic models, there is also uncertainty associated with the datasets that are input in the models, namely the modern ice load and post-LGM ice history. In the BedMachine ice thickness and bed elevation datasets, the mean error is ~100 m, although the pattern of uncertainty is strongly spatially heterogenous^14,15^. There are also uncertainties associated with the LGM-to-present ice history, for which the ICE-6G_C reconstruction used in this study is one of a number of possible models^1^. However, given the small magnitude of the post-LGM effects relative to the isostatic response to the removal of the far-better-constrained modern ice sheet loads (Fig. 2), these uncertainties in ice load history are unlikely to have significant implications for the overall isostatic response.

### Rebounded topographies

**Figure 5:**
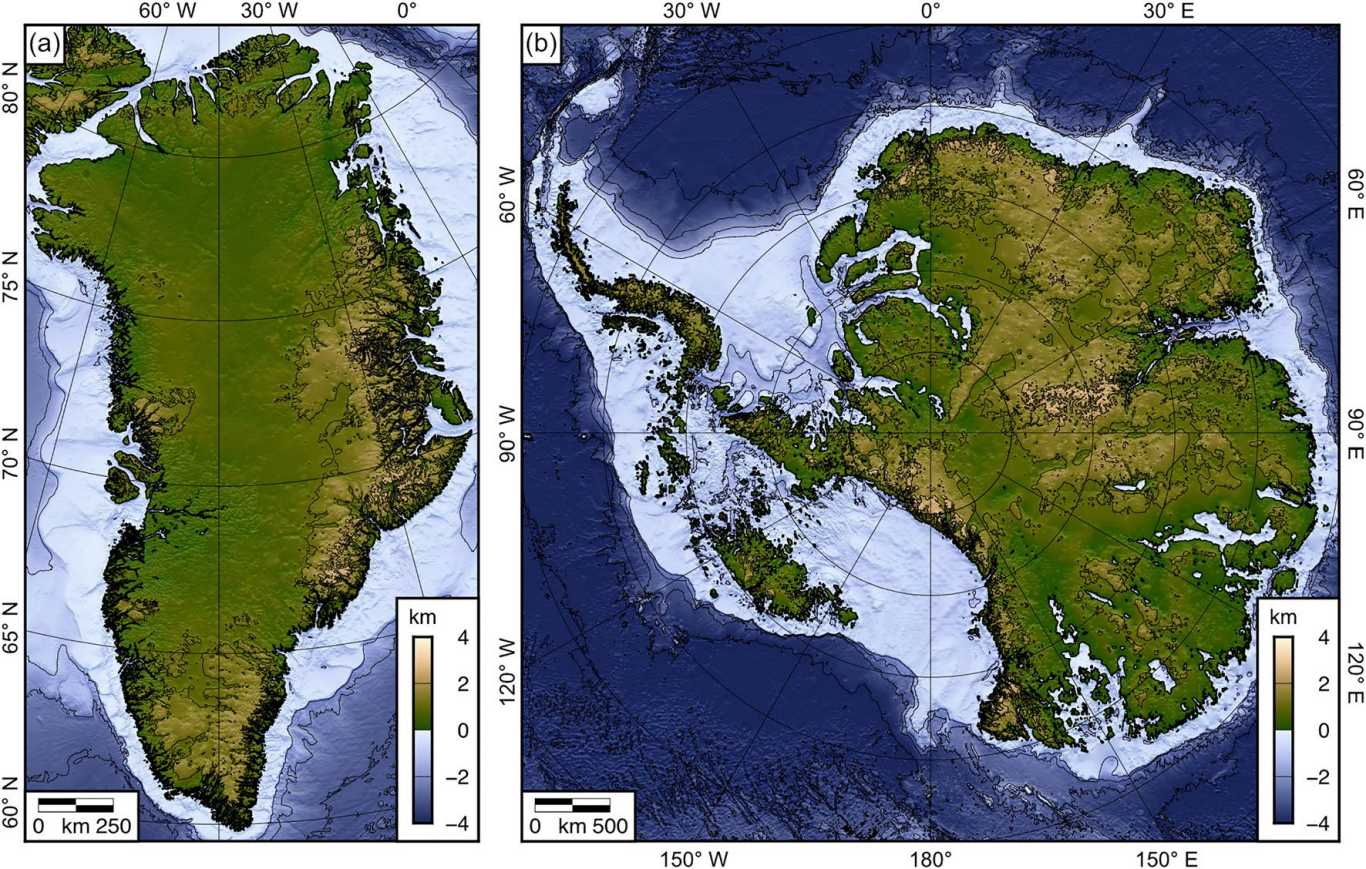
Fully rebounded bed elevations for Greenland and Antarctica. (**a**) Rebounded topography of Greenland. (**b**) Rebounded topography of Antarctica. Rebounded elevations are computed by summing the modern bed topographies and the total isostatic responses for the laterally variable *T*_*e*_ scenarios. Contour interval is 1 km. Elevations are relative to hypothetical global mean sea level in an ice-free world. Figure created using Generic Mapping Tools v.6 (https://www.generic-mapping-tools.org)^18^.

The total isostatic response fields (Fig. 3a,b) can be readily added to the respective BedMachine digital elevation models to generate a fully-equilibrated ice-free bed topography for Greenland and/or Antarctica (Fig. 5). Because the total isostatic response includes the deformation of the solid surface and the changes in geoid height, the rebounded bed elevations are referenced to a hypothetical global mean sea level for an ice-free world. However, since we do not take into account thermosteric or ocean dynamic effects, this datum is not an exact description of global mean sea level prior to glacial inception. The total isostatic response fields may also be directly sampled onto profile or point data (e.g., geophysical survey tracks or ice core locations) to isostatically adjust bed elevation measurements for the complete removal of the overlying ice. The accompanying standard deviation grids (Fig. 4) provide an estimate of the uncertainty in the isostatic response and may therefore be used to guide future studies aiming to better constrain *T*_*e*_ in Antarctica and Greenland by aiding the identification of particular regions where the magnitude of the flexural response is particularly sensitive to Earth structure.

## Discussion

The isostatic response to ice unloading in Greenland and/or Antarctica has been computed in a number of previous studies, typically as an intermediate step as part of a wider geoscientific analysis^5,10–12^. The grids presented in this study represent an improvement on previous calculations because they account for a number of factors that are often neglected, including (i) lateral heterogeneity in *T*_*e*_, (ii) a correction for post-LGM disequilibrium, and (iii) iterative computation of the isostatic effect of water loading. For example, the low *T*_*e*_ values that prevail in West Antarctica result in shorter-wavelength variability in isostatic uplift than in models that assume a uniformly high *T*_*e*_ for all of Antarctica^5,10^. Our isostatic response calculations also use the most recent high resolution modern ice thickness compilations^14,15^, and can be readily updated alongside future releases of improved ice thickness (and Earth structure) data products.

The elevation of the Greenlandic and Antarctic bed under ice-free conditions has a number of important applications. For example, the distribution of terrain situated below sea level under ice-free conditions (Fig. 5) may be used to predict which areas might have been flooded during ice-free times and in turn constrain the extent and connectivity of continental seaways and potential depocentres of marine sediment. The distribution of marine bed and/or inferred sediment coverage can potentially help to guide the selection of boundary conditions in numerical ice dynamical models such as basal sliding coefficients^35^. Fully-rebounded bed elevation (Fig. 5) is also an important parameter to consider when planning for potential recovery by sub-ice drilling of basal sedimentary material, which may contain vital records of palaeo-climate, -environmental conditions, and -ice extent^36,37^.

Finally, quantification of the ice-free elevation of Greenland and Antarctica is key for understanding the contributions of crustal compensation and mantle dynamics to the support of the present-day topography^9^. We note that these isostatic response grids are relevant for adjusting the bed topography only for the effects of ice (and water) (un)loading, and not the landscape-modifying effects of erosion, sedimentation, tectonic deformation, and lithosphere heating/cooling^38,39^. Instead, these updated adjustments for ice unloading represent the first stage in the reconstruction of a pre-glacial landscape, and as such may be integrated into future generations of palaeo-topographic and -bathymetric reconstructions.

## Conclusion

In this study we used recent compilations of ice thickness and lithospheric effective elastic thickness to compute the total equilibrated isostatic response to the complete unloading of the Greenland and Antarctic Ice Sheets. We envisage that these newly constructed grid files of the total isostatic response and the associated uncertainty can be used to correct bed elevations for the isostatic effects of ice loading at individual point locations, along geophysical survey tracks, and across wider regions up to and including the entirety of the landmasses. The gridded data products, which are openly accessible, will thus be of value to studies focussing on (e.g.,) ice sheet dynamics, landscape evolution, hydrological flow routing and drainage patterns, palaeo-environments, subglacial geology, tectonics, and geodynamics in the polar regions.

## Data Availability

The assembled grid files of the total isostatic response to ice sheet removal and associated uncertainty for Greenland and Antarctica developed in this study are available at the U.S. National Science Foundation Arctic Data Center (https://doi.org/10.18739/A2280509Z). The dataset contains NetCDF standard format files for both Greenland and Antarctica. The spatial projection, extent, and resolution of the total isostatic response grid files are equivalent to those of the BedMachine bed elevation models^14,15^.
